# Long-Term Effect of Semaglutide on the Glomerular Filtration Rate Slope in High-Risk Patients with Diabetic Nephropathy: Analysis in Real-World Clinical Practice

**DOI:** 10.3390/pharmaceutics17070943

**Published:** 2025-07-21

**Authors:** Enrique Luna, Álvaro Álvarez, Jorge Rodriguez-Sabiñón, Juan Villa, Teresa Giraldo, Maria Victoria Martín, Eva Vázquez, Noemi Fernández, Belén Ruiz, Guadalupe Garcia-Pino, Coral Martínez, Lilia Azevedo, Rosa María Diaz, Nicolas Roberto Robles, Guillermo Gervasini

**Affiliations:** 1 Nephrology Department, Badajoz University Hospital, 06006 Badajoz, Spain; enriquelh@unex.es (E.L.); juan.villa@salud-juntaex.es (J.V.); teresa.giraldo@salud-juntaex.es (T.G.); mariavictoria.martin@salud-juntaex.es (M.V.M.); 2 Department of Medical Biosciences, School of Medicine, University of Extremadura, 06006 Badajoz, Spain; 3 Institute of Molecular Pathology Biomarkers, University of Extremadura, 06006 Badajoz, Spain; 4 Don Benito-Villanueva Hospital, Nephrology Department, 06400 Don Benito, Spain; alvaro.alvarez@salud-juntaex.es (Á.Á.); jorgea.rodriguez@salud-juntaex.es (J.R.-S.); 5 Mérida Hospital, Nephrology Department, 06800 Mérida, Spain; eva.vazquez@salud-juntaex.es (E.V.); noemi.fernandez@salud-juntaex.es (N.F.); anabelen.ruiz@salud-juntaex.es (B.R.); 6 Zafra Hospital, Nephrology Department, 06300 Zafra, Spain; guadalupe.garciap@salud-juntaex.es (G.G.-P.); coral.martinez@salud-juntaex.es (C.M.); 7 Llerena Hospital, Nephrology Department, 06900 Llerena, Spain; lilia.oliveira@salud-juntaex.es (L.A.); rosa.diaz.campillejo@salud.madrid.org (R.M.D.); 8 Department of Medical & Surgical Therapeutics, School of Medicine, University of Extremadura, 06006 Badajoz, Spain

**Keywords:** semaglutide, chronic kidney disease, diabetes, SGLT2 inhibitors, estimated glomerular filtration rate, eGFR slope

## Abstract

**Background:** Semaglutide, a GLP-1 receptor agonist, has shown promising nephroprotective effects in clinical trials, though real-world data on its long-term impact on renal function in high-risk diabetic nephropathy patients remain scarce. **Methods:** We conducted a multicenter, retrospective observational study involving 156 patients with type 2 diabetes and chronic kidney disease (CKD) treated with subcutaneous semaglutide between 2019 and 2023. Inclusion required an eGFR > 15 mL/min/1.73 m^2^ or albuminuria > 30 mg/g and at least two years of follow-up. The primary outcome was the change in eGFR slope after semaglutide initiation. Subgroup analyses were performed based on baseline eGFR, albuminuria, and SGLT2i co-treatment. **Results:** In the whole study population, the median eGFR slope significantly improved from −3.29 (IQR 7.54) to −0.79 (IQR 6.01) mL/min/1.73 m^2^/year post-treatment (*p* < 0.001). Multiple linear regression showed a hazard ratio for the effect of semaglutide on the eGFR slope of 4.06 (2.43–5.68), *p* < 0.001. In patients with baseline eGFR < 60 mL/min/1.73 m^2^, the slope improved from −3.77 to −1.01 (*p* < 0.0001), while patients on concurrent SGLT2i therapy saw slope changes from −2.96 to −0.37 (*p* < 0.0001). Patients with albuminuria 30–1000 mg/g also improved from −2.96 to −0.04 (*p* < 0.0001); however, those > 1000 mg/g did not show a significant change (*p* = 0.184). Semaglutide also reduced BMI (*p* = 0.04), HbA1c (*p* = 0.002), triglycerides (*p* = 0.001), CRP (*p* = 0.003), and GGT values (*p* = 0.004). **Conclusions:** In real-world practice, semaglutide significantly attenuated renal function decline in high-risk diabetic patients, particularly those with advanced CKD or concurrent SGLT2i therapy. These findings support its nephroprotective role beyond glycemic control.

## 1. Introduction

Diabetic nephropathy (DN) is one of the main complications of diabetes mellitus that affects almost 40% of type 2 diabetic patients. It is known that DN implies a high risk of deterioration of kidney function with a significant annual drop in the estimated renal glomerular filtration rate (eGFR). This deterioration is influenced by poor chronic glycemic control, high levels of albuminuria, inflammatory phenomena, hyperfiltration and poorly controlled hypertension. In this context, renin–angiotensin–aldosterone axis inhibitors (RAASi) and sodium-glucose cotransporter 2 inhibitors (SGLT2i) have traditionally shown nephroprotective properties [1,2,3], helping reduce the eGFR slope decline. SGLT2i also have natriuretic effects, reduce albuminuria and decrease intraglomerular pressure, reducing hyperfiltration in patients with diabetic nephropathy. Recently, glucagon-like peptide-1 receptor agonists (GLP-1 RA) have also been shown to be nephroprotective [4,5,6]. Semaglutide, which belongs to this class of drugs, has shown a beneficial impact on body mass index (BMI), abdominal fat, hepatic steatosis [7] and inflammatory phenomena [8,9,10] in the general population. In addition, its use may result in cardiovascular and renal protection in patients with kidney disease [11,12].

Semaglutide was first shown to display nephroprotective properties in the SUSTAIN trial [13], a study primarily designed to evaluate protection against cardiovascular events, but that secondarily used robust criteria (reduction in the probability of requiring dialysis or the doubling of creatinine levels) to demonstrate nephroprotection. However, recent studies have concluded that to analyze a renal protective effect in a drug vs. placebo design using robust criteria requires expensive and very lengthy trials involving many patients [14,15]. Consequently, the evaluation of the eGFR slope with a follow-up of at least 3 years has been proposed as an adequate method to assess drug effects [16,17]. In this regard, -post hoc studies of semaglutide have found a significant reduction in the eGFR slope compared to placebo [18]. Recently, the FLOW trial demonstrated a nephroprotective effect of semaglutide in patients with chronic kidney disease (CKD) and type 2 diabetes on the eGFR slope. Supplementary Table S1 shows a summary of clinical trials evaluating renal effects of this drug.

To the best of our knowledge, no real-life studies have yet analyzed the effect of semaglutide on the eGFR slope in the mid-to-long term, nor has the effect of the combined semaglutide/SGLT2i treatment been properly determined. Hence, in the present work, we have aimed to fill this research gap by examining the impact of semaglutide on the eGFR slope, as well as to establish the effect of the addition of this drug to patients already being treated with SGLT2i.

## 2. Materials and Methods

### 2.1. Study Design

This was a multicenter, retrospective, observational study carried out in the nephrology services of five Spanish hospitals. The recruitment period was from January 2019 to October 2023 and patients were enrolled after signing a written informed consent. This was an observational cohort study approved by the Bioethics Committee of the institution. The inclusion criteria were patients aged over 18 years with type 2 diabetes mellitus treated with subcutaneous (SC) semaglutide, who were followed up in nephrology consultations and presented with an eGFR (calculated with the CKD EPI formula) > 15 mL/min/1.73 m^2^ or albuminuria > 30 mg/g of creatinine in urine after at least 3 tests. A minimum of one annual determination of serum creatinine and eGFR was needed during the follow-up, which was required to be longer than 2 years. Patients aged under 18 years, renal transplant recipients, patients taking part in other clinical trials, and those with both eGFR > 60 mL/min/1.73 m^2^ or albuminuria < 30 mg/g were excluded from the study.

### 2.2. Procedures

All the patients had received at least two oral antidiabetic medications prior to the introduction of semaglutide. Their glycemic control was closely tracked during follow-up and their antihypertensive and lipid-lowering treatment was intensified. Data from medical records were retrieved up to 4 years before administering semaglutide to study eGFR slopes prior to the treatment. Semaglutide was started at a dose of 0.25 mg SC per week, for 4 weeks. The dose was then increased to 0.5 mg/week for another 4 weeks, and then 1 mg/week. After starting treatment with semaglutide, an array of anthropometric and clinical parameters were obtained at 6 months, 1, 2, 3, and 4 years, namely weight, BMI, glycosylated hemoglobin A1c (HbA1c), lipid profile, high-sensitivity C-reactive protein (CRP), gamma-glutamyl transpeptidase (GGT), the urinary albuminuria/creatinine ratio (UACR), and eGFR, whose slope was considered the main outcome variable of the study, as we assumed that the introduction of semaglutide has low acute effects on eGFR [19].

### 2.3. Statistical Analysis

Renal function trajectory over time and decline in renal function was estimated as the slope of the individual linear regression line (B coefficient) of eGFR over the follow-up time, expressed as ± mL/min/1.73 m^2^/year with 95% confidence intervals (CI) in parenthesis. Negative or positive values of this parameter indicated renal disease progression or renal function improvement, respectively.

Parametric and non-parametric tests were used for the comparisons of continuous variables depending on the data distribution, whilst the chi-squared test was used to compare categorical variables. Multiple linear regression models adjusted by relevant covariates were used to assess the effect of semaglutide on the eGFR slope. Descriptive statistics are presented as mean ± standard deviation for continuous variables and as count and percentages for categorical variables. Nonparametric variables are presented as median and interquartile range (IQR). Statistical significance was established at a two-sided *p*-value < 0.05. All statistical analyses were performed using SPSS software (version 25.0, IBM Corp., Armonk, NY, USA).

## 3. Results

A total of 156 patients were included in the study with a mean follow-up of 942.3 ± 355.4 days. The average age of the enrolled patients was 64.3 ± 15.2 years, of whom 68% were men. Fifty-one percent of the patients had an eGFR between 30 and 60 mL/min/1.73 m^2^ and 72% had an eGFR between 60 and 15 mL/min/1.73 m^2^ (median = 35.8 IQR 17.1 mL/min/1.73 m^2^). Mean proteinuria was close to 1000 mg/g creatinine but presented a marked interindividual variability. Thirty-eight percent of the patients had macroalbuminuria (UACR > 300 mg/g), although values only exceeded 1000 mg/g in 17% of the cases. At the beginning of treatment with semaglutide, some 90% of the patients were using RAASi and 70% had already been treated with SGLT2i. Twenty-nine percent of the patients were also taking mineralocorticoids receptor antagonists (MRA) (Table 1).

### 3.1. Impact of Semaglutide Treatment on eGFR Slope

**Figure 1:**
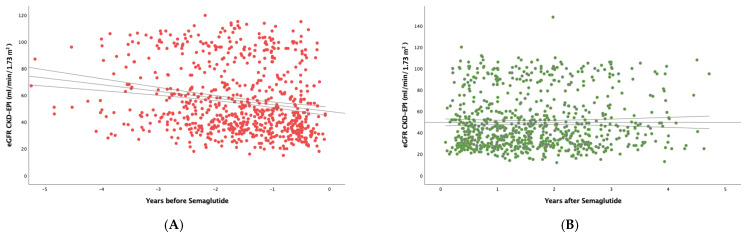
Estimated glomerular filtration rate (eGFR) values of all patients before (**A**) and after (**B**) starting semaglutide treatment. Trend line with 95% confidence interval is shown.

To study the real effect of semaglutide on renal function, we first determined in the whole study population the eGFR slope before implementing treatment with semaglutide, obtaining a median (IQR) value of −3.29 (7.54) mL/min/1.73 m^2^/year (Figure 1A). After the administration of the drug (Figure 1B), the eGFR slope significantly increased to −0.79 (6.01) mL/min/1.73 m^2^ per year (*p* < 0.001).

Multiple linear regression models adjusted by relevant covariates, namely use of SGLT2i, RAASi, MRA and albuminuria revealed that the effect of semaglutide on the eGFR slope showed a hazard ratio (HR) of 4.06 (2.43–5.68), *p* < 0.001.

**Figure 2:**
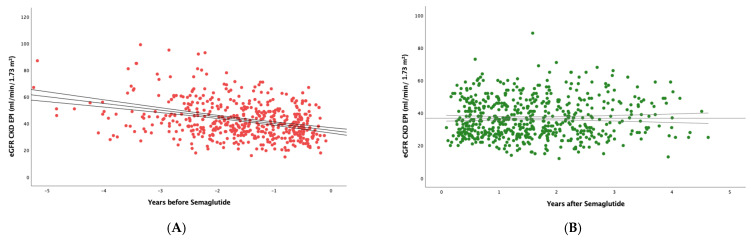
Estimated glomerular filtration rate (eGFR) values before (**A**) and after (**B**) treatment with semaglutide in patients with eGFR < 60 L/min/1.73 m^2^. Trend line with 95% confidence interval is shown.

Figure 2 shows that, in the subset of patients with eGFR <60 mL/min/1.73 m^2^, semaglutide had an even greater effect, as the slope went from −3.77 (6.88) to −1.01 (5.29) mL/min/1.73 m^2^/year; (*p* < 0.0001).

**Figure 3:**
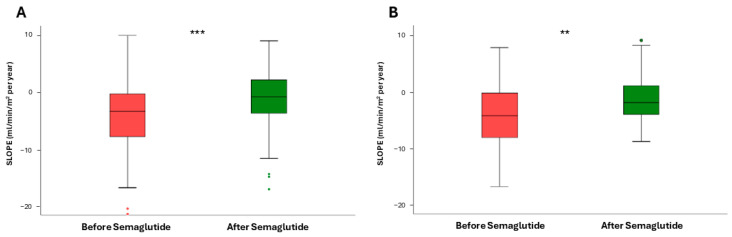
Mean values of estimated glomerular filtration rate (eGFR) slopes before and after treatment with semaglutide in patients who were already using SGLT2i (**A**) and in those who were not (**B**). ** *p* = 0.01, *** *p* < 0.0001.

Next, to assess the impact of the dual semaglutide/SLGT2i therapy, we then restrained the analysis to patients already taking SGLT2i. After the introduction of semaglutide, the slope varied from −2.96 (7.27) to −0.37 (5.87) mL/min/1.73 m^2^/year, *p* < 0.0001 (Figure 3A). In patients who were not on SGLT2i (Figure 3B), the slope also stabilized with respect to the values before the intervention −4.25 (8.07) vs. −1.91 (5.37) mL/min/1.73 m^2^/year (*p* = 0.01). However, the difference in the slope increment between both groups, i.e., patients on dual therapy and those only on semaglutide, was not statistically significant (*p* = 0.805).

**Figure 4:**
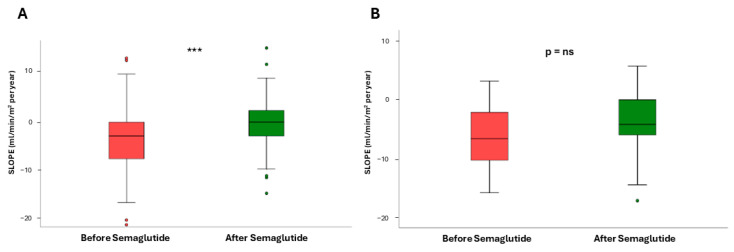
Impact of semaglutide on the estimated glomerular filtration rate (eGFR) slope in patients with urinary albumin-to creatinine ratio (UACR) = 30–1000 mg/g (**A**) and patients with UACR > 1000 mg/g (**B**). Mean values are shown. *** *p* < 0.0001; ns, not significant.

We finally analyzed changes in the eGFR slope according to the different degrees of albuminuria. Patients with UACR = 30–1000 mg/g had a median slope of −2.96 (7.22) before semaglutide and −0.04 (5.34) mL/min/1.73 m^2^/year after semaglutide (*p* < 0.0001, Figure 4A). For those patients with UACR > 1000 mg/g (Figure 4B) the increase was much smaller, as eGFR values went from −6.61 (9.09) to −4.24 (6.07) mL/min/1.73 m^2^/year (*p* = 0.184). As in the case of dual vs. monotherapy, there were no significant differences when we compared the slope increments obtained with the treatment for patients with low and high UACR values (*p* = 0.274).

### 3.2. Effect of Semaglutide on Other Clinical Parameters

Table 2 shows that BMI values decreased significantly during the follow-up (from 29.1 ± 4.8 to 27.3 ± 4.4, *p* = 0.04), as did HBA1c levels (from 7.3 (2.1) to 6.6 (1.9) %, *p* = 0.002). An improvement in triglyceride levels was also evident (from 219.2 ± 142.7 to 162.2 ± 9.5). In the same manner, there were subclinical inflammatory phenomena at baseline, as measured by CRP, which were significantly reduced at the end of the study (*p* = 0.003). The degree of hepatic steatosis, monitored by determining the plasma GGT [20,21], also showed a significant reduction (*p* = 0.004). In contrast, no significant changes were observed in proteinuria or albuminuria, although it should be noted that patients at baseline presented with an extremely high variability, and that most patients had low albuminuria at the beginning of the study.

## 4. Discussion

**Figure 5:**
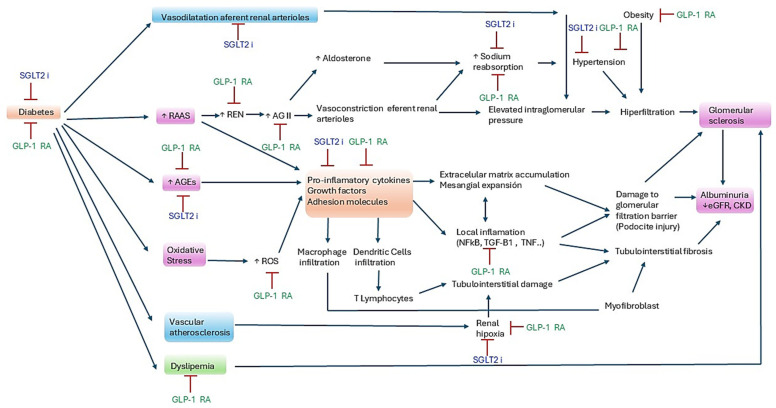
Synergistic nephroprotective mechanism of GLP-1 RA and SGLT2i. Red lines denote inhibition. RAAS, Renin–Angiotensin–Aldosterone system; AGEs, advanced glycation end products; ROS, reactive oxygen species; REN, renin; AGII, angiotensin II; TFG-B1, transforming growth factor B1; TNF, tumor necrosis factor; NK-kB, nuclear factor kappa B; eGFR, estimated glomerular filtration rate; CKD, chronic kidney disease.

CKD associated with type II diabetes mellitus causes significant cardiovascular morbidity and mortality. It is also associated with a high risk of progressive worsening of kidney function and a high risk of end-stage kidney disease and dialysis. In turn, this decline in renal function leads to a greater risk of cardiovascular disease and death [22]. This is basically the reason why therapies that lead to stability or improvement in kidney function also improve the general prognosis of these patients. In this regard, SGLT2i and GLP-1 RA present a variety of synergistic mechanisms that make them useful in patients with diabetic nephropathy. SGLT2i have natriuretic effects, reduce albuminuria and decrease intraglomerular pressure, reducing hyperfiltration [23]. In the same manner, semaglutide not only increases sodium excretion while decreasing albuminuria and hyperfiltration but, in addition, it reduces body fat and the production of cytokines involved in the progression of CKD [24]. Figure 5 depicts these and other putative mechanisms.

The SUSTAIN-6 trial demonstrated that SC semaglutide reduces the risk of major renal events, when considered as a composite variable, by reducing macroalbuminuria [13]. However, the study did not show significant differences compared with placebo regarding the need for dialysis or the doubling of serum creatinine. These studies, whose primary objective is cardiovascular safety, are generally too short to assess renal protection using robust and longer-term endpoints, hence the use of eGFR slope in this and other studies [25].

On the other hand, a meta-analysis by Sattar et al. on the nephro- and cardioprotective effects of GLP-1 RA [12] reported a neutral effect on the overall kidney function. It should be noted, however, that the potency and pharmacokinetic characteristics of the different GLP-1 RA used in the trials differed significantly [26], which could have generated some bias. In this regard, we exclusively used SC semaglutide in our study to avoid variations in bioavailability, which are inherent to oral formulations. Recently, the FLOW trial demonstrated a nephroprotective effect of semaglutide in patients with CKD and type 2 diabetes, as the risk of a primary outcome event was 24% lower, and the mean annual eGFR slope was less steep in the semaglutide group [27]. However, SGLT2i and MRA agents were not yet approved when the trial began, and hence the ability of the study to assess the effect of combination therapy with semaglutide was very limited. In our analysis, we studied patients with type 2 diabetes mellitus and CKD with a marked decline in kidney function and high risk of kidney function deterioration. After starting semaglutide, we found that the eGFR slope improved significantly in the mid-to-long term. These findings agree with post hoc studies from the SUSTAIN trial demonstrating that SC semaglutide had a particularly beneficial effect on the eGFR slope decline when compared to placebo, an effect that was greater than that of oral semaglutide or liraglutide, another GLP-1 RA [18].

To the best of our knowledge, there are no previous reports studying the effect of SC semaglutide on the eGFR slope in real-life practice, let alone with a mean follow-up of 3 years and in a sizeable cohort of patients with reduced renal function. Remarkably, in our study, it was precisely the patients with more compromised renal function who benefited the most from the introduction of semaglutide, which highlights the potential of this drug to improve cardiovascular function in a high-risk population such as CKD patients by stabilizing (and even improving) their kidney function.

It has also been shown that a combined SGLT2i/GLP-1AR therapy in a diabetic population with CKD stage 3/4 (eGFR = 15–60 mL/min/1.73 m^2^) and high risk of rapid deterioration of kidney function, may have a synergistic cardio- and nephroprotective effect [28]. Our study, which included patients with a steeply negative eGFR slope, and that therefore had higher morbidity levels and a greater risk of kidney failure [22], confirms that nephroprotection was evident in patients on dual therapy. Even though in absolute numbers this group finished with a far lower eGFR slope than patients on semaglutide alone, nonparametric tests yielded no statistical differences between both populations. In any case, it is remarkable that patients on dual therapy reached an annual decline of only −0.37 mL/min, when the average decline in healthy subjects from 40 years of age onwards is approximately −1 mL/min/year [29,30]. Two very recent real-life studies have examined the combined effect of GLP-1 RA/SGLT2i, although they did not measure its impact on the eGFR slope. Jhu et al. retrospectively analyzed patients with eGFR > 60/mL/min/year, for 5 years, with the GLP-1 RA/SGLT2i group having significantly lower risk of major adverse kidney events [31], whilst Chu et al. investigated the effectiveness of GLP-1 RA compared with basal insulin among adults treated with mainstay SGLT2i therapy for T2D and CKD. The study showed that eGFR was reduced, although significantly less in the GLP-1 RA group than in the basal insulin cohort [32]. Similar results were obtained by post hoc studies [33]. In contrast, the effect of this combined SGLT2i/GLP-1AR treatment could not be assessed in former seminal trials such as SUSTAIN 6 (with only 2% of the patients treated with SGLT2i) [13] or FLOW (15.6% patients on dual treatment) [27]. In any case, it should be noted that, in our study, patients who were not treated with SGLT2i also presented a significant improvement in the negative slope of eGFR after being administered semaglutide. A post hoc study from FLOW trial has recently confirmed our results [34].

With regard to the role of albuminuria, our data showed that patients with lower levels of albuminuria (<1000 mg/g) presented better slopes than those with UACR values above 1000 mg/g. However, only 17% of our patients presented with UACR > 1000 mg/g and hence, drawing conclusions for this specific subgroup is adventurous. This selection bias could also explain why we did not observe a significant improvement in albuminuria values during the follow-up, as opposed to the data reported by the SUSTAIN trial.

Finally, we observed a significant reduction in the BMI values of our patients after the introduction of semaglutide. Weight loss in the population with diabetic nephropathy is extremely beneficial, not only because it reduces cardiovascular risk inherent to obesity, but also because it decreases hyperfiltration, which protects against the development of further sclerosis of the glomeruli, therefore reducing progression of kidney damage [35]. In addition, the reduction in adipose tissue produced by semaglutide decreases its ability to generate harmful cytokines and hormones [36,37,38,39]. Moreover, we observed several other additional beneficial effects after the long-term treatment with semaglutide, such as a decrease in inflammatory markers and improvement in hepatic steatosis, which are involved in insulin resistance and the poor cardiovascular prognosis of these patients [8,10,40,41].

This study has several limitations. First, because of its observational and retrospective nature, a causal relationship cannot be formally established based on our findings; second, follow-up data for the time SGLT2i had been used prior to starting semaglutide were not available, which could have provided more context for the interpretation of the eGFR slope before the introduction of the GLP-1 RA; third, the improvement in eGFR could also be the result of an unquantified loss of lean mass after weight loss; and, finally, the study included a relatively small number of Caucasian patients; therefore, larger and more diverse cohorts are needed to confirm the results presented herein. In particular, studies including the black population, where the eGFR must be adjusted, would be most informative.

In conclusion, the findings of this real-life study indicate that the use of semaglutide in diabetic patients with a high risk of CKD can prevent the progressive decline in renal function they would otherwise invariably experience. Our findings indicate that semaglutide may be used for patients with diabetic nephropathy, especially in advanced stages. In the future, it would be valuable to evaluate its protective effect in clinical trials with obese/overweight CKD patients without diabetes. In the same manner, the combined use of SGLT2i and semaglutide should definitely be studied with dedicated clinical trials in diabetic individuals with some degree of CKD, with a particular focus on those patients with stage 3 and 4 and high BMI values.

## Figures and Tables

**Table 1:** Demographic and clinical characteristics at baseline. Values shown are means ± standard deviation or percentages.

Variable	
BMI	29.1 ± 4.8
Age (years)	64.3 ± 15.2
Sex (female)	32.9%
Cardiovascular background	38.2%
eGFR (mL/min/1.73 m^2^)	43.1 (IQR 38.2)
HBA1c (%)	7.3 (IQR 2.1)
CRP (mg/L)	5.3 ± 7.7
LDL-cholesterol (mg/dL)	77.4 ± 30.3
Triglycerides (mg/dL)	219.2 ± 142.7
GGT (mg/dL)	49.3 ± 72.9
Proteinuria (mg/g Cr)	362.7 (IQR 866.6)
Albuminuria (mg/g Cr)	145.1 (IQR 499.1)
Albuminuria (30–300 mg/g Cr)	61.8%
Albuminuria (300–1000 mg/g Cr)	21.4%
Albuminuria (>1000 mg/g Cr)	16.8%
Use of ACEIs/ARA II	88.8%
Use of SGLT2i	70.2%
Use of MRA	30.3%

eGFR estimated glomerular filtration rate; HbA1c, glycosylated hemoglobin; CRP, C-reactive protein; GGT, gamma-glutamyl transpeptidase; ACEIs, angiotensin-converting enzyme inhibitors; ARA II, angiotensin II receptor antagonists; SGLT2i, sodium-glucose transport protein 2 inhibitors; MRA, mineralocorticoid receptor antagonist.

**Table 2:** Clinical parameters of patients at baseline and 6 months, 1, 2, and 3 years after introduction of semaglutide. Numbers are shown as mean (standard deviation) or median (interquartile range, IQR), depending on data distribution.

	Baseline	6 Month	1 Year	2 Years	3 Years	* *p*
BMI	29.1 ± 4.8	28.4 ± 4.8	28.1 ± 4.7	27.3 ± 3.4	26.3 ± 3.4	0.04
eGFR (mL/min/1.73 m^2^)	43.1 (IQR 38.2)	45.5 (36.1)	42.1 (35.2)	42.5 (38.2)	42.4 (37.1)	0.573
HbA1c (%)	7.3 (IQR 2.1)	6.7 (1.5)	6.5 (1.3)	6.8 (1.9)	6.6 (1.9)	0.002
CRP (mgr/L)	5.3 ± 7.7	3.9 ± 3.9	3.1 ± 3.1	3.4 ± 5.4	3.2 ± 0.6	0.003
LDL-c (mg/dL)	77.4 ± 30.3	66.3 ± 23.2	70.7 ± 28.7	66.2 ± 26.2	66.1 ± 26.3	0.066
Triglycerides (mg/dL)	219.2 ± 142.7	190.9 ± 109.4	183.5 ± 149.9	164.1 ± 82.4	162 ± 95.5	0.001
GGT (mg/dL)	49.3 ± 72.9	42.8 ± 62.7	38.2 ± 42.6	37.1 ± 41.7	37.1 ± 40.1	0.004
Proteinuria (mg/g Cr)	362.7 (IQR 866.6)	337.1 (652.2)	320.2 (996.1)	328.5 (799.3)	320 (690.1)	0.570
Albuminuria (mg/g Cr)	145.1 (IQR 499.1)	114.2 (444.1)	99.5 (661.4)	98.5 (518.2)	99.1 (520.2)	0.584
CKD stage 1–2	27.9%	29.3%	28.9%	28.8%	31.1%	0.571
CKD stage 3	50.6%	49.3%	49.3%	46.2%	42.2%	
CKD stage 4	21.5%	21.3%	21.8%	25%	26.7%	

BMI, body-mass index; eGFR, estimated glomerular filtration rate; HbA1c, glycosylated hemoglobin; CRP, C-reactive protein; GGT, gamma-glutamyl transpeptidase; Cr, creatinine; CKD, chronic kidney disease. * *p*-value for the comparison of baseline vs. end of study.

## Data Availability

Source data for this study are openly available at the Figshare repository: https://doi.org/10.6084/m9.figshare.29108321.v1.

## Supplementary Materials

The following supporting information can be downloaded at: https://www.mdpi.com/article/10.3390/pharmaceutics17070943/s1, Table S1. Summary of clinical trials evaluating renal effects of semaglutide.

## Abbreviations

The following abbreviations are used in this manuscript:

CKD	Chronic kidney disease
DN	Diabetic nephropathy
eGFR	Estimated glomerular filtration rate
GLP-1AR	Glucagon-like peptide-1 receptor agonist
RAASi	Renin–angiotensin–aldosterone axis inhibitors
SGLT2i	Sodium-glucose cotransporter 2 inhibitors
UACR	Urine albumin-to-creatinine ratio
