# Supplementary material for: Long-Term Effect of Semaglutide on the Glomerular Filtration Rate Slope in High-Risk Patients with Diabetic Nephropathy: Analysis in Real-World Clinical Practice
Source: Pharmaceutics. 2025 Jul 21;17(7):943. doi: 10.3390/pharmaceutics17070943 (PMC12298743; doi:10.3390/pharmaceutics17070943)
Supplement: Supplementary file 1 [file pharmaceutics-17-00943-s001.zip › pharmaceutics-3713635-supplementary.pdf]

Supplementary Table S1. Summary of clinical trials evaluating renal effects of semaglutide.

	**Treatment**	**Patients enrolled**	**Type of patients**	**Median follow-up**	**Risk analysis for kidney-specific outcome**	**Difference in annual eGFR slope (ml/min/1.73 m2 /year)**
SUSTAIN-6 trial [13]	Subcutaneous Semaglutide vs placebo	3297	Type II DM	2 years	HR 0.64 (0.46-0.88)	-
Apperloo et al. [33]	Subcutaneous or oral Semaglutide vs placebo	6480	Type II Diabetes and high cardiovascular risk. P*ost hoc* pooled analysis of SUSTAIN 6 and Pioneer 6 trials	2 years	-	0.59 (0.29-0.89)
FLOW trial [27]	Subcutaneous Semaglutide vs placebo	3533	Type II DM and CKD.	3.4 years	HR 0.79 (0.66-0.94)	1.16 (0.86-1.47)
Mann et al. [34]	Subcutaneous Semaglutide ± SGLT2i vs placebo	3533	Post-hoc FLOW trial. Type II DM and CKD.	3.4 years	-	0.75 (95% CI: −0.01, 1.50) and 1.25 (95% CI: 0.91, 1.58) with or without SGLT2i use, respectively

DM, diabetes melitus; CKD, chronic kidney disease; SGLT2i, sodium-glucose cotransporter 2 inhibitors; HR, hazard ratio
